# Exogenous Melatonin Regulates Puberty and the Hypothalamic GnRH-GnIH System in Female Mice

**DOI:** 10.3390/brainsci12111550

**Published:** 2022-11-15

**Authors:** Zixuan Chen, Lina Si, Weihan Shu, Xin Zhang, Chenyang Wei, Meng Wei, Luyang Cheng, Zhihong Chen, Yuebing Qiao, Songhe Yang

**Affiliations:** 1 Department of Human Anatomy, Chengde Medical University, Chengde 067000, China; 2 Department of Immunology, Chengde Medical University, Chengde 067000, China; 3 Faculty of Graduate Studies, Chengde Medical University, Chengde 067000, China

**Keywords:** melatonin, puberty, GnRH, GnIH, Kisspeptin, Neuropeptide Y, Proopiomelanocortin, hypothalamic–pituitary–gonadal axis

## Abstract

In recent years, the age of children entering puberty is getting lower and the incidence of central precocious puberty is increasing. It is known that melatonin plays an increasingly important role in regulating animal reproduction, but the specific role and mechanism of melatonin in regulating the initiation of puberty remain unclear. The purpose of the current study was to investigate the effect of subcutaneous melatonin injection on pubertal development in female mice and its mechanism of action. Female mice that were 22 days old received 1 mg/kg doses of melatonin subcutaneously every day for 10, 15 and 20 days. The vaginal opening was checked daily. Hematoxylin and eosin (HE) stain was used to determine the growth of the uterus and ovaries. Enzyme-linked immunosorbent assay (ELISA) was used to determine the levels of follicle-stimulating hormone (FSH), gonadotropin-inhibiting hormone (GnIH), and gonadotropin-releasing hormone (GnRH) in serum. By using RT-PCR and Western blotting, the mRNA and protein expression of the hypothalamus GnRH, GnIH, Kisspeptin (Kp), Proopiomelanocortin (POMC), Neuropeptide Y (NPY), as well as G protein-coupled receptor 147 (GPR147) were identified. The findings demonstrated that melatonin could suppress ovarian follicle and uterine wall growth as well as delay vaginal opening, decrease serum levels of GnRH and FSH and increase levels of GnIH. Melatonin increased GnIH and GPR147 expression in the hypothalamus in comparison to the saline group, while decreasing the expression of GnRH, Kisspeptin, POMC, and NPY. In conclusion, exogenous melatonin can inhibit the onset of puberty in female mice by modulating the expression of hypothalamic GnRH, GnIH, Kisspeptin, POMC and NPY neurons and suppressing the hypothalamic–pituitary–gonadal axis.

## 1. Introduction

Gonadotropin-releasing hormone (GnRH) is a peptide hormone consisting of 10 amino acids and is found mainly in neural tissue [1]. The hypothalamus’s increased GnRH production causes puberty to start [2]. Gonadotropins, which act on the gonads to drive sperm and egg formation, are secreted by the anterior pituitary gland after GnRH induces their release [3,4]. In addition, initiation of pubertal development may also be regulated by a variety of substances, including Melatonin (MLT) [5], Kisspeptin (Kp) [6,7], Proopiomelanocortin (POMC), Neuropeptide Y (NPY), endogenous opiate like substance, Leptin, etc [8]. These signals are integrated with each other to promote the initiation and development of adolescence. Gonadotropin-inhibiting hormone (GnIH), a hypothalamic neuropeptide that suppresses the production of gonadotropin in quail, was initially discovered by researchers in 2000 [9]. GnIH homologues RFamide-related peptides 1 and 3 (RFRP-1 and -3) were subsequently discovered in mammals and primates, which prevent the release and synthesis of gonadotropin in mammals and birds while also inhibiting GnRH-induced gonadotropin secretion [9,10]. The hypothalamic-pituitary-gonadal axis (HPGA) is regulated and modulated by GnRH and GnIH, and its control of gonadotropin secretion plays a crucial role in the mammalian reproductive system.

Melatonin is a neurohormone produced mostly in the pineal gland and is released mainly during the dark period of the circadian rhythm [11,12,13], named melatonin because it causes the skin of frogs to turn white [14]. In many areas of neuroendocrine and physiological function, melatonin is a key regulator, including seasonal changes and circadian rhythm [15], maturation [5], aging [16], neuroendocrine [17,18] and cardiovascular [19]. Melatonin has been used extensively in humans for hypnosis, anti-aging, and anti-cancer treatments [20,21,22]. Melatonin is a key player in the release of reproductive hormones, oocytes development, luteal functions, ovulation, and early embryo growth in female mammals [23,24].

Previous research has demonstrated that a progressive drop in blood melatonin levels during puberty’s commencement may aid in triggering the hypothalamus to release GnRH, so triggering the reproduction axis and accelerating the beginning of puberty [25]. In recent years, the results show that different doses of melatonin have different effects on different species. Male albino wistar rats’ beginning of puberty is accelerated by exogenous melatonin, and this impact is stronger in the younger animals [26]. By breaking down endocrine barriers, melatonin can accelerate the start of puberty and improve ewes’ reproductive capabilities [27]. Young female mice were given a substantial dose of melatonin (15 mg/kg) intraperitoneally, which expedited the start of puberty [28]. However, melatonin implantation before puberty in ewes delayed the onset of puberty by 4 weeks [29]. Short photoperiod or melatonin treatment can delay puberty in male Bulgarian hamsters [30]. Collectively, these research advances suggest that melatonin is a key factor affecting mammalian reproductive and developmental processes. The mechanisms behind these regulatory effects, however, remain poorly understood. This study’s objective was to verify the regulatory effect of subcutaneous injection of physiological doses of melatonin on pubertal development in female mice, and whether its mechanism regulates the reproductive axis by acting on the hypothalamic GnRH-GnIH system.

## 2. Materials and Methods

### 2.1. Animals

Purchase of 78 Kunming (KM) mice (weight, 10~12 g) without specific pathogens from Beijing Huafukang Company Biotechnology Co., Ltd. (certificate no. 11401300067446, Beijing, China). With enough food and water available, all mice were kept in a space with a twelve hour circadian rhythm, a constant temp of 24 °C and 40% humidity. The Experiment Animal Welfare Ethics Committee of Chengde Medical College accepted all animal related research method, and ensured that they were carried out in accordance with Chengde Medical University’s ethical standards (SYXK(JI)2022-002).

### 2.2. Animal Treatment and Grouping

After couple of days of acclimatization, the KM mice were separated into three groups at random: the 10-day group (n = 26); the 15-day group (n = 26); and the 20-day group (n = 26). The melatonin experimental group (MLT; n = 13) and the normal saline group (NS; n = 13) were included in each group. Due to the increased sensitivity of melatonin receptors in the afternoon, in the melatonin group, melatonin (M5250-1G; Merck Sigma Biologicals, St. Louis, MI, USA) was injected subcutaneously 1 mg/kg daily in the afternoon starting at 22 days old. Melatonin was soluble in anhydrous ethanol, then the ethanol mass concentration was diluted with saline to a final level of 5%. Similar amounts of saline were injected into the normal saline group. Every morning, each group of mice’s vaginal opening (VO) was routinely checked. The mice were weighed after 10, 15 and 20 days of injection, respectively, followed by intraperitoneal sodium pentobarbital 0.5% anesthesia (40 mg/kg); blood was collected from the heart, the uterus and ovaries removed and preserved in 4% paraformaldehyde. After cervical dislocation execution, the hypothalamus was removed and stored at −80 °C for backup.

### 2.3. Puberty Onset in Female Mice

The vaginal opening is often used as an external marker to judge the sexual development of female mice. The vaginas opening dates of mice were observed and recorded daily. The procedure was as follows: place the female on the cage, hold its head and waist, turn its tail over to expose her vulva, and record the time of opening the vagina.

### 2.4. Pathological Examination of the Uterus and Ovary

Mice’s uterus and ovaries were preserved in 4% paraformaldehyde for 48 h. To dehydrate, alcohol was applied gradually. The ovaries and uteri were subjected to xylene treatment before being fixed in paraffin and cut into 4 μm sections. At 50 °C, the parts were dried in an oven. After that, the sections were dewaxed with xylene and hydrated with gradient alcohol. Hematoxylin and eosin was then used to stain the sections. Sections were sealed with neutral resin after being dried with alcohol and made transparent with xylene. We took pictures of ovarian sections at 200× magnification and uterine sections at 40× magnification under a light microscope, and observed the development of follicles and uterus in each group.

### 2.5. Hormonal Analysis of Serum

Each mouse’s cardiac blood was collected and centrifuged for fifteen minutes at 3000× *g* as well as at 4 °C. The serum was then obtained and kept at −20 °C for later analysis. GnRH, follicle-stimulating hormone (FSH), and GnIH serum hormone levels were analyzed using ELISA kits in accordance with the manufacturer’s instructions. Mouse GnRH (EEL0071C; Elabscience Biotechnology, Inc., Wuhan, China), mouse FSH (EELM0511C; Elabscience Biotechnology, Inc., Wuhan, China), and mouse GnIH ELISA kits (ml764151; Shanghai Enzymelinked Biotechnology Co., Ltd, China) were used to measure the levels of these hormones in the mouse serum. The sensitivity of the GnRH kit was 9.38 pg/mL, and the intra- and inter-assay coefficients of variation were <10%. The sensitivity of the FSH kit was 0.94 ng/mL, and the intra- and inter-assay coefficients of variation were <10%. The sensitivity of the GnIH kit was 0.1 mIU/mL, and the intra- and inter-assay coefficients of variation were <10% and <15%.

### 2.6. Real-Time Fluorescence Quantitative Polymerase Chain Reaction (RT-PCR) Assay

The MiniBEST Universal RNA Extraction Kit (TaKaRa Biotechnology Co., Ltd, Dalian, China) was used to obtain all total RNA from the hypothalamus, and the RNA concentration was determined using an Ultra-micro nucleic acid protein detector. RNA was reverse transcribed into complementary DNA (cDNA) using a kit from Takara Biotechnology Co., Ltd. In accordance with the manufacturer’s instructions, a complete of 2 μL of cDNA template were exposed to the Polymerase chain reaction in a 25 μL reaction process using SYBR Premix Ex Taq II (TaKaRa Biotechnology Co., Ltd.). For amplification and quantitation, a real-time PCR instrument (Bio-Rad Laboratories, Hercules, CA, USA) was employed. The initial denaturation at 95 °C for 30 s, the denaturation at 95 °C for 5 s, the annealing at 60 °C, the extension for 30 s, and the repeating of 40 cycles make up the amplification cycle. The GAPDH gene was used as the control. The mRNA expression levels was then calculated using the 2^−ΔΔCt^ approach. Sangon Biotech Co., Ltd. designed the primers (Shanghai, China). Table 1 displays the sequence of the primers.

### 2.7. Western Blot Analysis

The RIPA lysis buffer (R0020100ML; Beijing Solarbio Science & Technology Co., Ltd, China) was used to lyse the hypothalamus tissue, which was then centrifuged at 12,000 rpm for 20 min at 4 °C. The Protein Assay by BCA kit (PC0020500; Beijing Solarbio Science & Technology Co., Ltd.) was used to measure the concentration of protein. Equal mass of protein (30 μg) from the lysates of the hypothalamus tissue were divided on 12% SDS-PAGE and then transported to the PVDF membrane (IPVH00010; EMD Millipore, Inc., Shanghai, China). The blocking liquid of TBS and Tween with skim milk was applied to the PVDF membrane and left on for two hours at 37 °C. Rabbit anti-Kiss-1 (1:500; sc-15400; Santa Cruz Biotechnology, Inc., Dallas, TX, USA), anti-GnRH (1:1000; A8424; ABclonal Technology, Inc., Wuhan, China), anti-POMC (1:500; BS7477; Bioworld Technology, Inc., USA), anti-Neuropeptide Y (1:1000; 11976T; Cell Signaling Technology, Inc., Danvers, MA, USA), anti-NPFF-1 receptor (1:500; A422561; Beijing Biosynthesis Biotechnology Co., Ltd, Beijing, China), and rabbit anti-GAPDH (1:1000; 14C10; Cell Signaling Technology, Inc., USA) were the primary antibodies used. The primary antibody was incubated in the refrigerator at 4 °C for one night and TBS-Tween 20 was washed three times. HRP-labeled secondary antibody (1:8000; AS014; ABclonal Technology, Inc., Wuhan, China) was added, sourced from goat anti-rabbit, and incubated at 37 °C for one hour. The membrane was washed three times and then the proteins were visualized with ultra-sensitive ECL chemiluminescent substances (Biosharp Technology, Inc., Beijing, China). The average gray values were analyzed using Image J analysis software (version 1.46r).

### 2.8. Statistical Analysis

Software called SPSS v22.0 was used for data analysis (IBM Corp, USA). Independent samples t-test and two-way ANOVA were both used to analyze differences between two and three groups, respectively. Mean ± standard deviation was used to express the results. Vaginal opening rate was analyzed using Fisher’s probabilities in 2 × 2 table. *p* > 0.05 were regarded as non-significant, while *p* < 0.05 as statistically significant.

## 3. Results

### 3.1. Effects of Melatonin on Vaginal Opening Rate in Female Mice

**Figure 1:**
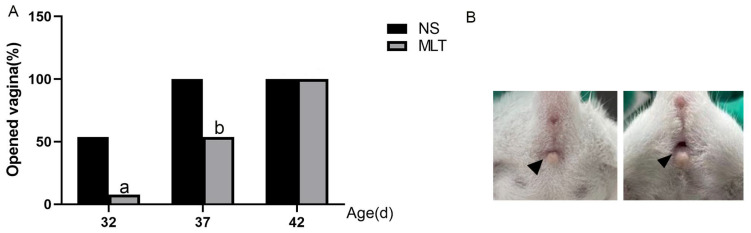
Melatonin’s effects on female mice’s vaginal opening rate (n = 13). (**A**) Vaginal opening rate. (**B**) Vaginal photo. (**Left**): vagina closed; (**Right**): vagina open (Black triangle). a: *p* < 0.05 vs. 32 dNS; b: *p* < 0.05 vs. 37 dNS; 32 d, 32 days; 37 d, 37 days; 42 d, 42 days; NS, normal saline; MLT, melatonin.

The estrus status of the mice was studied following melatonin injection in order to research the impact of melatonin on prepubescent mice. In mice, the vaginal opening indicates the start of puberty. According to the analysis, the MLT group’s vaginal openness rate was considerably lower than the NS group’s at 32 and 37 days old: 7.69% vs. 53.85% (NS) (*p* < 0.05); 53.85% vs. 100% (NS) (*p* < 0.05), and in both groups, the vaginal openness rate at 42 days old was 100% (*p* > 0.05). The findings demonstrated that melatonin prevented female mice from going through puberty (Figure 1).

### 3.2. Effects of Melatonin on Body Weight in Female Mice

**Figure 2:**
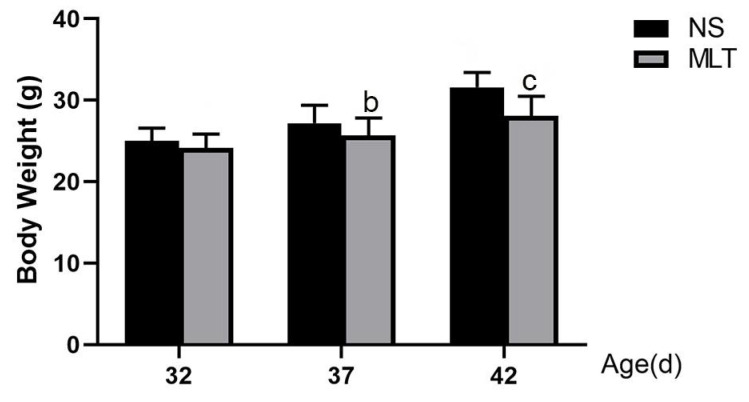
Melatonin’s effects on female mice’s body weight (n = 13). b: *p* < 0.05 vs. 37 dNS; c: *p* < 0.05 vs. 42 dNS; 32 d, 32 days; 37 d, 37 days; 42 d, 42 days; NS, normal saline; MLT, melatonin.

The reproductive performance of mammals is closely related to the level of nutrition. We speculate that melatonin may influence puberty by affecting physical development. Therefore, we measured the body weight of each group on the anesthesia sampling day. The body weight was 24.15 ± 1.76, 25.73 ± 1.75 and 28.07 ± 1.93 g after 10, 15 and 20 days of subcutaneous melatonin injection, respectively, while the body weights of the NS groups were 25.03 ± 1.70, 27.20 ± 1.70 and 31.55 ± 1.90 g, respectively. Mice’s body weight gradually grew with age. At 37 and 42 days old, the MLT group’s body weight considerably decreased in comparison to the NS group (*p* < 0.05; Figure 2).

### 3.3. Effects of Melatonin on Ovarian and Uterine Development

**Figure 3:**
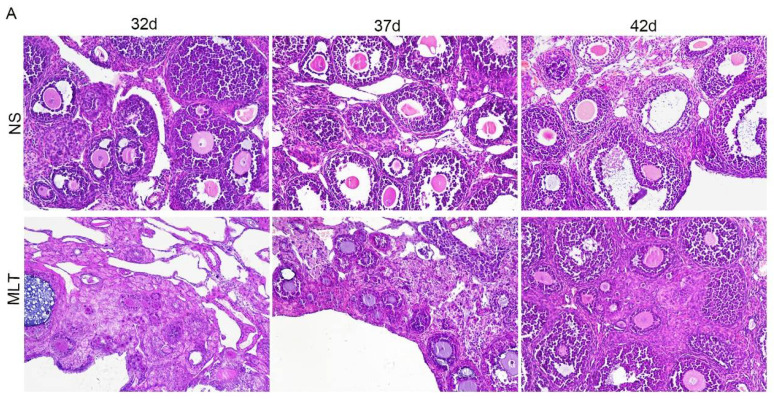

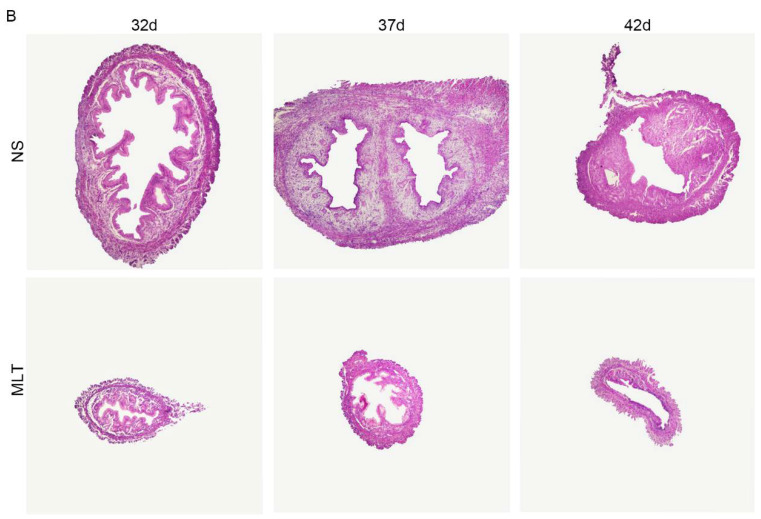
Melatonin’s effects on female mice’s follicular maturation and uterine wall thickness (n = 13). (**A**) Development of follicles in each group by HE staining, magnification, 200×. (**B**) Development of uterus in each group by HE staining, magnification, 40×. 32 d, 32 days; 37 d, 37 days; 42 d, 42 days; NS, normal saline; MLT, melatonin.

To examine follicle development and the thicknesses of the wall of the uterus, HE was dyed into the mice’s ovaries and uteri. The majority of the follicles in the NS group of mice were tertiary follicles, mature follicle along with a few secondary follicles. The number of primary and primordial follicles in the ovaries of the mice in the NS group was essentially minimal. The MLT group of mice’s ovaries, however, included many primordial and primary follicles. The MLT group’s follicles increasingly matured as they grew older, and their secondary and tertiary follicle counts increased (Figure 3). The uterine wall of mice in the MLT group was clearly thinner than that of the NS group. With increasing age, the uterine wall thickness in the NS group gradually thickened, and the MLT group was basically unchanged (Figure 3).

### 3.4. Effects of Melatonin on the Levels of the Female Mouse Serum Hormones GnRH, FSH, and GnIH

**Figure 4:**
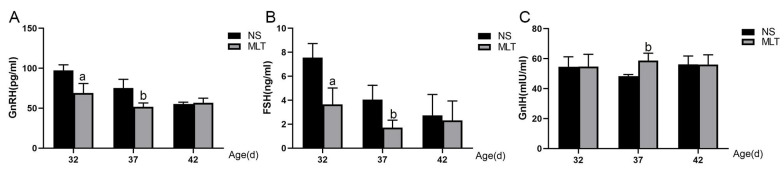
Melatonin’s effects on female mice’s serum GnRH, FSH and GnIH hormone levels (n = 13). (**A**) Melatonin decreased serum GnRH concentration. (**B**) Melatonin decreased serum FSH concentration. (**C**) Melatonin increased serum GnIH concentration. a: *p* < 0.05 vs. 32 dNS; b: *p* < 0.05 vs. 37 dNS; 32 d, 32 days; 37 d, 37 days; 42 d, 42 days; NS, normal saline; MLT, melatonin.

ELISA was used to measure the levels of GnRH, FSH, and GnIH in the serum (Figure 4). After 10, 15, and 20 days of subcutaneous melatonin injection, the serum GnRH concentrations were 69.08 ± 11.83, 51.79 ± 4.79 and 56.65 ± 5.95 pg/mL, respectively, while the equivalent GnRH concentrations in the NS groups were 97.32 ± 7.00, 75.47 ± 10.76 and 55.37 ± 2.14 pg/mL, respectively. When compared to the NS groups, the GnRH concentrations in the MLT group were considerably lower at 32 and 37 days old (*p* < 0.05). At 42 days old, there was no difference in the two groups. (*p* > 0.05; Figure 4).

The concentration of serum FSH was 3.64 ± 1.38, 1.71 ± 0.62 and 2.32 ± 1.62 ng/mL after 10, 15 and 20 days of subcutaneous melatonin injection, respectively, while in the NS groups, the comparable FSH levels were 7.55 ± 1.18, 4.04 ± 1.21 and 2.73 ± 1.75 ng/mL. When compared to the NS groups, the MLT group’s FSH concentrations at 32 and 37 days old were considerably lower (*p* < 0.05). At 42 days old, there was no difference in the two groups (*p* > 0.05; Figure 4).

The concentration of serum GnIH was 54.78 ± 8.13, 58.72 ± 4.91 and 56.10 ± 6.49 mIU/mL after 10, 15 and 20 days of subcutaneous melatonin injection, respectively, while in the NS groups, the comparable GnIH levels were 54.60 ± 6.66, 48.33 ± 1.10 and 56.16 ± 5.58 mIU/mL. GnIH concentrations were considerably higher in the MLT group compared to the NS group at 37 days old (*p* < 0.05), but at 32 and 42 days old, there were no significant statistical differences (*p* > 0.05; Figure 4). These findings imply that subcutaneous melatonin injection decreases GnRH and FSH secretion and increases GnIH secretion in the blood of KM mice, and that the three hormones exhibit comparable trends at various times.

### 3.5. Effects of Melatonin on the Expression of Reproductive-Related Neuronal mRNA in Female Mice’s Hypothalamus

RT-PCR analysis was carried out to show whether melatonin has a regulatory influence on Kiss-1, GnRH, POMC, NPY, GnIH, and GPR147 in the hypothalamus. After 10, 15 and 20 days of melatonin injections, the gene expression levels of Kiss-1 in the mice’s hypothalamus were 0.41 ± 0.03, 0.84 ± 0.06 and 0.91 ± 0.05, respectively; the gene expression levels of GnRH in the mice’s hypothalamus were 0.16 ± 0.01, 0.30 ± 0.01 and 1.27 ± 0.21, respectively; the gene expression levels of POMC in the mice’s hypothalamus were 0.91 ± 0.04, 0.68 ± 0.08 and 0.78 ± 0.03, respectively; the gene expression levels of NPY in the mice’s hypothalamus were 0.25 ± 0.01, 0.64 ± 0.02 and 0.73 ± 0.05, respectively; the gene expression levels of GnIH in the mice’s hypothalamus were 4.70 ± 0.15, 0.35 ± 0.01 and 1.15 ± 0.18, respectively; the gene expression levels of GPR147 in the mice’s hypothalamus were 1.17 ± 0.15, 0.76 ± 0.05 and 1.11 ± 0.06, respectively.

**Figure 5:**
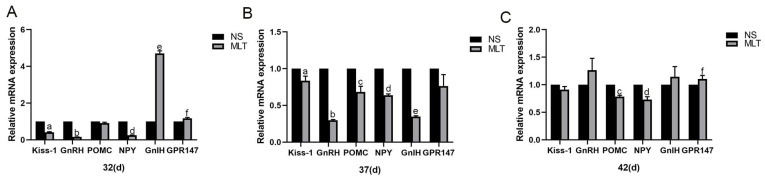
Melatonin’s effects on the expression of reproductive-related neuronal mRNA in the female mice’s hypothalamus (n = 13). (**A**) At 32 days old. (**B**) At 37 days old. (**C**) At 42 days old. a, b, c, d, e, f: *p* < 0.05 vs. the NS group; 32 d, 32 days; 37 d, 37 days; 42 d, 42 days; NS, normal saline; MLT, melatonin.

The findings showed that, at 32 days old, mice in the MLT group had significantly lower mRNA expression levels of Kiss-1, GnRH and NPY than mice in the NS group (*p* < 0.05), greater levels of GnIH and GPR147 mRNA than the NS group (*p* < 0.05), and no major differences in the expression level of POMC mRNA (*p* > 0.05; Figure 5). At 37 days old, the MLT group’s expression of the genes for Kiss-1, GnRH, POMC, NPY, and GnIH was considerably lower than that of the NS group (*p* < 0.05), whereas the expression level of the gene for GPR147 had not changed appreciably (*p* > 0.05; Figure 5). When compared to the NS group at 42 days old, the MLT group’s level of POMC and NPY mRNA was considerably reduced (*p* < 0.05), while the MLT group’s level of GPR147 mRNA was higher (*p* < 0.05), but the level of Kiss-1, GnRH, and GnIH mRNA did not alter significantly (*p* > 0.05; Figure 5). According to these findings, subcutaneous melatonin injection reduces the levels of expression of Kiss-1, GnRH, POMC, and NPY mRNA and increases the expression levels of GnIH and GPR147 mRNA in the mice’s hypothalamus, with similar trends of changes in these three mRNAs at different time points. 

### 3.6. Effects of Melatonin on the Expression of Reproductive-Related Neuronal Protein in Female Mice’s Hypothalamus

**Figure 6:**
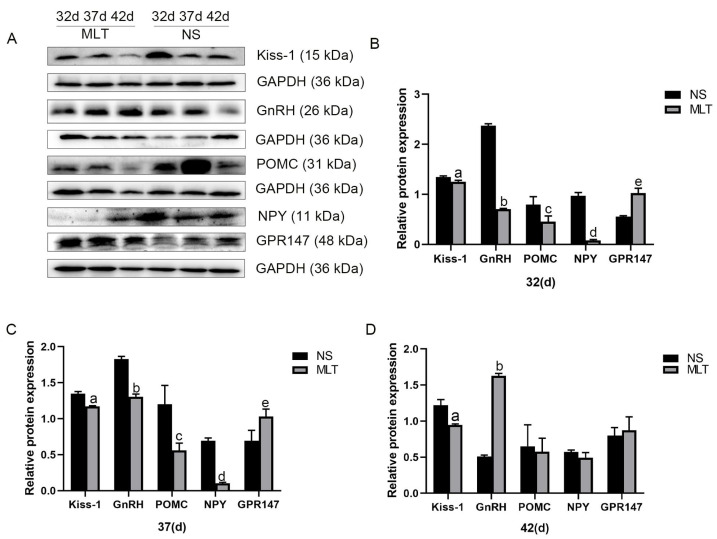
Melatonin’s effects on the expression of neuronal proteins associated to reproduction in the mice’s hypothalamus and its potential mode of action (n = 13). (**A**) Protein expression levels of Kiss-1, GnRH, POMC, NPY and GPR147 after subcutaneous injection of melatonin were detected via western blot analysis. (**B**) At 32 days old. (**C**) At 37 days old. (**D**) At 42 days old. a, b, c, d, e: *p* < 0.05 vs. the NS group. 32 d, 32 days; 37 d, 37 days; 42 d, 42 days; NS, normal saline; MLT, melatonin.

Western blot research was carried out to show whether melatonin has a regulatory influence on Kiss-1, GnRH, POMC, NPY, and GPR147 in the hypothalamus. The percentage of the destination protein’s gray value to the internal reference GAPDH is used to represent levels of expression. Kiss-1 protein expression levels in the mice’s hypothalamus were 1.26 ± 0.03, 1.17 ± 0.01 and 0.95 ± 0.01, respectively, after 10, 15, and 20 days of melatonin injections, whereas they were 1.35 ± 0.02, 1.35 ± 0.03 and 1.22 ± 0.08 in the NS groups. Significantly less Kiss-1 protein was expressed in the MLT groups than in the NS groups (*p* < 0.05; Figure 6). 

The GnRH protein expression levels in the mice’s hypothalamus were 0.71 ± 0.01, 1.31 ± 0.04 and 1.63 ± 0.03, respectively, after 10, 15 and 20 days of melatonin injections, whereas they were 2.37 ± 0.04, 1.38 ± 0.04 and 0.51 ± 0.02 in the NS groups. At 32 and 37 days old, the MLT groups’ expression of the GnRH protein was considerably lower than that of the NS groups (*p* < 0.05); at 42 days old, the MLT groups’ expression of the GnRH protein was considerably higher than that of the NS groups (*p* < 0.05; Figure 6).

The POMC protein expression levels in the mice’s hypothalamus were 0.46 ± 0.11, 0.56 ± 0.10 and 0.58 ± 0.19, respectively, after 10, 15 and 20 days of melatonin injections, whereas they were 0.79 ± 0.16, 1.20 ± 0.26 and 0.65 ± 0.30 in the NS groups. At 32 and 37 days old, the MLT groups’ expression of the POMC protein was considerably lower than that of the NS groups (*p* < 0.05); at 42 days old, there was no distinction between the two groups (*p* > 0.05; Figure 6).

The NPY protein expression levels in the mice’s hypothalamus were 0.08 ± 0.02, 0.10 ± 0.01 and 0.49 ± 0.07, respectively, after 10, 15 and 20 days of melatonin injections, whereas they were 0.97 ± 0.07, 0.69 ± 0.04 and 0.57 ± 0.03 in the NS groups. At 32 and 37 days old, the MLT groups’ expression of the NPY protein was considerably lower than that of the NS groups (*p* < 0.05); at 42 days old, there was no distinction between the two groups (*p* > 0.05; Figure 6).

The GPR147 protein expression levels in the mice’s hypothalamus were 1.03 ± 0.09, 1.03 ± 0.10 and 0.87 ± 0.19, respectively, after 10, 15 and 20 days of melatonin injections, whereas they were 0.55 ± 0.02, 0.69 ± 0.14 and 0.80 ± 0.11 in the NS groups. At 32 and 37 days old, the MLT groups’ expression of the GPR147 protein was considerably higher than that of the NS groups (*p* < 0.05); at 42 days old, there was no distinction between the two groups (*p* > 0.05; Figure 6). These findings imply that subcutaneous melatonin injection reduces the level of the Kiss-1, GnRH, POMC, and NPY proteins and enhances the level of the GPR147 protein in the mice’s hypothalamus, with similar trends of changes in these three proteins at various time periods. 

## 4. Discussion

Melatonin has multiple effects on the regulation of animal reproductive performance, mainly by acting on receptor sites in the HPGA. In seasonal breeding animals, melatonin exhibits inhibitory or promotive effects on reproductive performance. According to studies, melatonin inhibits reproduction in humans and long daylight breeding animals like cattle, rats, and other creatures. And that is secretory activity in the pineal gland is significantly increased during the progressively shorter daylight period, with higher melatonin concentrations and inhibition of synthesis and secretion of hypothalamic GnRH and pituitary gonadotropin [31]. Melatonin has direct effects on gonadal organs like the ovaries and testes in addition to controlling animal reproductive activity via the HPGA. Some researchers have reported that melatonin can inhibit testosterone secretion from the testes and estradiol secretion from the ovaries [32].

Despite research on melatonin’s function in animal reproduction, the specific mechanism of melatonin’s action during puberty remains unclear. The aim of this study was to investigate the mechanism by which melatonin affects the development of puberty in female mice. The experimental design was to give prepubertal 22-day-old female mice a daily afternoon subcutaneous injection of melatonin at a dose of 1 mg/kg. Firstly, we wanted to observe the effect of melatonin injections of different duration on puberty in mice. Secondly, since mice become reproductively active after 6 weeks of age, we wanted to observe the effect of melatonin on mice during early (32 days of age), late (37 days of age) and sexual maturity (42 days of age) of puberty. Therefore, we chose to inject melatonin for 10 days, 15 days and 20 days. The physiological dose of 1 mg/kg was selected based on the pre-experiments of different dose concentrations. Since the pineal gland secretes melatonin mainly at night, the injection of exogenous melatonin at this time does not increase the sensitivity of the receptors, and the sensitivity of the melatonin receptors is lowest in the morning, so we choose to inject exogenous melatonin in the afternoon. 

In mice, vaginal opening is an outward marker of the beginning of puberty, and gonadal development is an internal sign. It has been discovered that melatonin slows down the opening of the vagina in mice and prevents the growth of ovarian follicles and uterine. The mechanism of action of melatonin was further explored. The surrounding environment of this study is consistent, since the surrounding environment can influence the vaginal opening. Some studies suggest that body weight is a major factor controlling the onset of puberty [33]. Puberty was induced only when female mice reached a certain body weight. Each group of mice in this study had their body weight measured. The findings demonstrated that mice’s body weight continuously rose with age, and at 37 and 42 days of age, the melatonin group’s body weight considerably decreased in comparison to the normal saline group. It is suggested that melatonin delays puberty development in mice may be related to its inhibition of growth and development.

An essential neuropeptide produced in the hypothalamus called GnRH controls the release of gonadotropins in vertebrates. It acts as a connection between the endocrine and neurological systems [34]. It induces the production of pituitary gonadotropins (FSH and LH) by operating on the anterior pituitary gland that expresses GnRH-R [3,4], which have a great impact on pubertal development and gonadal and reproductive functions. GnIH is a neuropeptide inhibitor that acts directly on GnRH neurons in the hypothalamus and pituitary through the receptor NPFFR1 (also known as GPR147) to inhibit gonadotropin synthesis and release, thereby suppressing reproductive function [35,36]. NPFFR1 is justexpressed by some GnRH neurons, hence GnIH may control the GnRH axis via interacting with other interneurons, like Kisspeptin neurons. It has been demonstrated that the homologous peptide RFRP-3 nervous fiber of GnIH in mice can connect to the PeN of Kisspeptin neurons and the anterior ventral periventricular nucleus (AVPV) [37], and NPFFR1 is expressed by interneurons (such as Kisspeptin neurons), and GnIH can link to it to suppress the activity of GnRH neurons [35]. In the neurone of the hypothalamus, RFRP-3 can co-express with Kisspeptin and can connect to Kisspeptin directly [38]. Along with controlling reproduction, various researchs [10,36,39] have discovered that GnIH also controls eating behavior in vertebrates. In sheep, NPY, POMC and melanin concentrating hormone neurons are projected via GnIH nerve fibers. These neurons have a direct or indirect impact on eating, metabolism, GnRH activation, and gonadotropin secretion. They are found in the arcuate nucleus, a critical region in metabolism control and reproduction [8]. In 1996, Kiss-1 was already discovered in the melanoma cells [40], and adolescent rats had considerably higher levels of Kiss-1 mRNA expression [41,42]. After kisspeptin-10 was injected into the brain of sheep, large amounts of GnRH were observed in the cerebrospinal fluid. This is because Kisspeptin (the product encoded by Kiss-1) binds to G protein-coupled receptor 54. Research has shown the double-labeled in situ hybridization tests demonstrate that 77% of GnRH neurons co-express GPR54 mRNA [43]. Kisspeptin also directly stimulates LH release from the pituitary gland in female or male rats [44]. Kiss-1 is a key link in seasonal reproduction. In seasonally breeding animals, photoperiod can directly modulate Kiss-1 signaling via melatonin to drive the reproductive axis [45]. Kiss gene expression decreased after treatment with 10 nM melatonin in the rat hypothalamic clonal immortalized cell model rHypoE-8, however, RFRP-3 expression increased in rHypoE-7 cells [46]. NPY is a polypeptide containing 36 amino acids isolated from pig brain in 1982, which is closely related to the energy metabolism, growth and reproduction of animals. It has a strong appetite-promoting effect, is involved in regulating feeding and reproduction, stimulates feeding and inhibits gonadotropin release [47]. POMC is an important appetite-regulating peptide located in the arcuate nucleus. As a precursor protein, after being hydrolyzed by specific proteases, many small-molecule polypeptides related to food regulation and energy balance are generated. Studies have shown that POMC can inhibit food intake and stimulate reproduction [48]. Kisspeptin, NPY, and POMC neurons have been found in studies to direct operate on GnRH neurons and control GnRH release, altering reproductive function [49,50].

It has been shown that in photoperiodic quail species, melatonin produced by the eye and pineal gland acts through melatonin receptor mellc on hypothalamic GnIH neurons to stimulate the expression of GnIH [51]. Exogenous melatonin can exponentially increase GnIH release from the hypothalamus. Additionally, melatonin and GnIH also decreased testosterone release in vitro studies. These suggest that in the animal gonadal axis, especially in birds, animal reproductive activity is modulated by the interaction of melatonin and GnIH [52]. The reproductive state of Siberian hamsters is primarily driven by day length (photoperiod). Photoperiodic mammals rely on annual cycles of melatonin secretion by the pineal gland at night to drive their reproductive responses [53]. It has been shown that GnIH precursor mRNA expression and the number of immunoreactive positive cells were lower in Siberian hamsters in the short-day photoperiod (SD) than in the long-day photoperiod (LD). Melatonin inhibits the expression of GnIH precursor mRNA as well as GnIH polypeptide [54]. It was also found that the percentage of GnRH-ir neurons receiving GnIH-ir fiber terminations was reduced during the SD photoperiod. This pattern of GnIH expression and its relationship to seasonal changes in reproductive status suggests that short-term melatonin in LD stimulates GnIH expression to suppress high concentrations of luteinizing hormone, whereas long-term melatonin in SD suppresses GnIH expression to block GnRH stimulation [55]. This result is consistent with that of the Syrian hamster. The mechanism of differential action of melatonin on GnIH (RFRP) expression in different species is an issue that has not yet been elucidated.

The easiest to understand clinical effectiveness measures are serum hormone levels [56]. These findings show that melatonin has crossed the blood-brain barrier into the brain to inhibit the secretion of GnRH and FSH and promote the secretion of GnIH, and are consistent with previous studies. However, there were differences in hormone levels between the groups. It is speculated that the release of reproductive hormones is pulsatile and the frequency is difficult to grasp. In addition, there is a significant overlap in the levels of secretion between the phases influenced by the sexual cycle, so the results are sometimes difficult to determine. Suppression of gonadal development (ovaries and uterus) was observed in mice by HE staining due to decreased levels of GnRH and FSH secretion. Additionally, melatonin raised the expression levels of GnIH and GPR147 while decreasing the expression levels of hypothalamic Kiss-1, GnRH, POMC, and NPY. Melatonin has been reported to control energy balance and improve obesity by suppressing appetite-promoting polypeptide (NPY) in patients with osteoporosis [57]. Consequently, the current investigation indicated that melatonin suppresses appetite and controls weight gain by inhibiting NPY expression in the hypothalamus of mice. From the results, it can be found that the protein expression levels of these factors are different from the mRNA expression levels. The reason for the analysis may be that the target genes were detected at the transcriptional level by PCR technique. The amounts of gene and protein expression are not always positively associated, though, because the transition from transcription to translation is so difficult.

**Figure 7:**
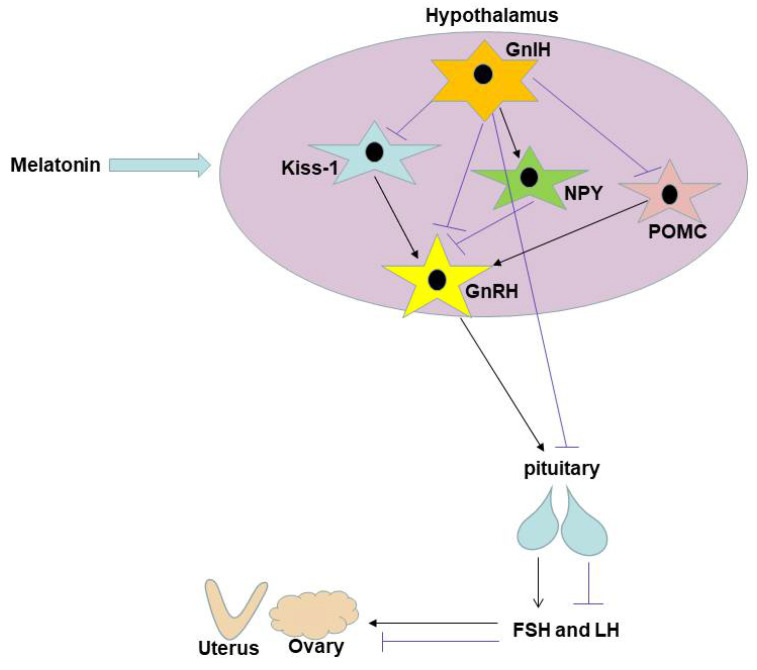
The melatonin mediating delayed puberty pattern diagram.

The results of this research show that subcutaneous melatonin inhibits the maturation of the pubertal stage in female mice. Previous studies have shown that intraperitoneal administration of high doses of melatonin (15 mg/kg) promotes the release of FSH and thus accelerates the initiation of puberty in mice. And the mRNA levels of GnRH and GnRHr, key genes in GnRH signaling in the hypothalamus that control FSH synthesis, were unchanged. It can be hypothesized that the positive effect of melatonin on FSH synthesis is not mediated by the hypothalamus, but is directly accelerated by acting on the pituitary gland to accelerate the onset of puberty [28]. Melatonin can cross the blood–brain barrier, so we choose to prolong melatonin receptor sensitivity by slowly absorbing it through subcutaneous injection. Our findings suggest that melatonin acts on GnRH neurons in the hypothalamus to decrease GnRH expression and release, which in turn stops the release of gonadotropins and the start of puberty. Melatonin may also act indirectly on Kiss-1, POMC and NPY neurons by acting on GnIH neurons and promoting GnIH expression and release, thereby preventing the secretion of GnRH and delaying the beginning of puberty (Figure 7). Whether melatonin inhibits reproduction by acting directly on Kiss-1, POMC and NPY neurons requires further investigation. It is possible that this result is different from the previous result due to the different injection method and dose. The current work suggests possible key points for the treatment of precocious puberty and provides evidence for a better understanding of the mechanisms of melatonin regulation in fertility.

## 5. Conclusions

In conclusion, subcutaneous administration of physiological doses of melatonin to prepubertal female mice delayed puberty and inhibited gonadal development, probably by regulating the expression of GnRH, kiss-1, GnIH, POMC and NPY neurons in the hypothalamus, thereby inhibiting the hypothalamic–pituitary–gonadal axis. This study provides a new key point for the treatment of children with precocious puberty.

## Figures and Tables

**Table 1:** Primers’ sequence.

Name of the Gene	Primer Sequence (5′-3′)	Amplification Fraction (bp)
	Forward:	
GnRH	ACTGCTGACTGTGTGTTTGGAAGG	136
	Reverse: TTCTGCCATTTGATCCACCTCCTTG	
	Forward:	
GnIH	CCCCAAGACACCCGCTGATTTG	123
	Reverse: CTCCTCGTTCGCTTTCCACCAG	
	Forward:	
GPR147	CGAGTCTGAACGAGAGTGATGCTG	80
	Reverse: CGGAGAGGAGTGCTGGTAGTAGG	
	Forward:	
Kiss-1	GCTGCTGCTTCTCCTCTGTGTC	119
	Reverse: GCGATTCCTTTTCCCAGGCATTAAC	
	Forward:	
POMC	TAGAGTTCAAGAGGGAGCTGGAAGG	141
	Reverse: CACCGTAACGCTTGTCCTTGGG	
	Forward:	
NPY	TGTGTTTGGGCATTCTGGCTGAG	117
	Reverse: TGAGATTGATGTAGTGTCGCAGAGC	
	Forward:	
GAPDH	GGTTGTCTCCTGCGACTTCA	183
	Reverse: TGGTCCAGGGTTTCTTACTCC	

## Data Availability

The datasets used and/or analyzed during the current study are available from the corresponding author on reasonable request.
